# Metabolomics and Integrative Omics for the Development of Thai Traditional Medicine

**DOI:** 10.3389/fphar.2017.00474

**Published:** 2017-07-18

**Authors:** Sakda Khoomrung, Kwanjeera Wanichthanarak, Intawat Nookaew, Onusa Thamsermsang, Patcharamon Seubnooch, Tawee Laohapand, Pravit Akarasereenont

**Affiliations:** ^1^Center of Applied Thai Traditional Medicine, Faculty of Medicine Siriraj Hospital, Mahidol University Bangkok, Thailand; ^2^Siriraj Metabolomics and Phenomics Center, Faculty of Medicine Siriraj Hospital, Mahidol University Bangkok, Thailand; ^3^Systems and Synthetic Biology, Department of Biology and Biological Engineering, Chalmers University of Technology Gothenburg, Sweden; ^4^Department of Biomedical Informatics, College of Medicine, University of Arkansas for Medical Sciences Little Rock, AR, United States; ^5^Department of Pharmacology, Faculty of Medicine Siriraj Hospital, Mahidol University Bangkok, Thailand

**Keywords:** metabolomics, analytical chemistry, herbal medicines, integrative omics, Thai traditional medicine

## Abstract

In recent years, interest in studies of traditional medicine in Asian and African countries has gradually increased due to its potential to complement modern medicine. In this review, we provide an overview of Thai traditional medicine (TTM) current development, and ongoing research activities of TTM related to metabolomics. This review will also focus on three important elements of systems biology analysis of TTM including analytical techniques, statistical approaches and bioinformatics tools for handling and analyzing untargeted metabolomics data. The main objective of this data analysis is to gain a comprehensive understanding of the system wide effects that TTM has on individuals. Furthermore, potential applications of metabolomics and systems medicine in TTM will also be discussed.

## Search methodology

Literature search procedure that we used to produce this manuscript is described below. The PubMed, Google Scholar, Web of Science database were searched with the terms of “metabolomics,” “traditional Thai medicine,” “metabolome analysis,” “herbal medicine” or “analytical chemistry,” “integrative data analysis” or “omic analysis” or “univariate analysis” or “multivariate analysis” or “omics in Chinese herbal medicine”. Search criteria were included original research articles, review articles, books, national reports that were published in English language only.

## Thai traditional medicine (TTM)

Traditionally, Thai traditional medicine (TTM) is defined as a holistic medicine that comprises both methods and practices. The TTM is heavily influenced by Buddhism. According to this religious belief, the human body is composed of four elements: earth, water, wind and fire, and an imbalance in one of these elements will lead to illness (Chokevivat and Chuthaputti, 2005). TTM consists of four different aspects: medical practice (diagnosis and treatment), pharmacy practice (the production and the use of herbal medicines), traditional midwifery and traditional Thai massage (Akarasereenont et al., 2015). A number of theories and hypotheses used in TTM have accumulated from several eras including Sukhothai to Ayutthaya (1350–1767), Thonburi (1767–1782) and the early period of Rattanakosin (Bangkok) in 1782–1851 (Chuthaputti and Boonterm, 2010). This knowledge was built based on the historical experiences, traditional wisdom, and ancient medical textbooks that were passed down and developed by each generation to practictioners. To promote TTM in the national healthcare system, the Thai government has continued to fully support TTM research, particularly since 1997 in the area of medicinal plants. This national strategic plan resulted in widespread research on herbal medicines, with most of the research being focused on the preclinical study of single herbs. This action has led to the development of several databases of herbal medicine such as, Medplant online (http://www.medplant.mahidol.ac.th) by the Faculty of Pharmacy at Mahidol University, and Thaicrudedrug (http://www.thaicrudedrug.com) by the Faculty of Pharmaceutical Sciences at Ubon Ratchathani University.

Although research and development efforts involving TTM have continued to increase, the use of herbal medicines for the treatment of illnesses in Thailand is relatively low compared to modern medicine. This is mostly due to the lack of clinical evidence, especially in the aspect of efficacy and safety of the various herbal medicines. Therefore, additional effort is needed for the deep investigation of herbal medicines at both molecular and phenotypic levels.

## Metabolomics

The advent of high-throughput technology known as “omics” has proven to be very useful across multiple areas of biology. Among these, metabolomics has shown to be a promising tool to describe the phenotypes in a dynamic context. Metabolomics is the area of study that seeks to identify and quantify the complete set of metabolites in a given organism (Nicholson et al., 1999; Fiehn, 2001). Typically, metabolites are defined as small molecules (<1 kDa) that are intermediates or products of metabolic reactions (Holmes et al., 2008). Metabolome analysis normally consists of a series of several steps that include sample preparation, measurement and data analysis (Villas-Bôas et al., 2006; Mushtaq et al., 2014). In metabolomics, quenching is a process used to stop metabolite turnover, especially during the sampling and sample preparation steps. The process is highly effective for most of the primary metabolites such as, amino acids, sugars, organic acids or carbohydrates. Secondary metabolites, on the other hand, which include a group of metabolites that are derived from three families such as, phenolics, alkaloids, and terpenes and steroids (Bourgaud et al., 2001), typically have a much slower turnover rate and are more chemically stable than primary metabolites, elminating the need to quench during sample preparation. These latter groups of metabolites are often of more interest for use in traditional medicine (Kennedy and Wightman, 2011). After quenching, samples are extracted, which typically involves a wide range of organic or inorganic solvents such as, methanol (Kanchanapoom et al., 2001; Nakamura et al., 2008; Sawasdee et al., 2009; Tripatara et al., 2012; Padumanonda et al., 2014), ethanol (Sutthanut et al., 2007; Thiengsusuk et al., 2013), ethyl acetate (Shimokawa et al., 2013), or hexane (Lu et al., 2009) depending upon the metabolites of interest. There are numerous extraction methods available, e.g., classical solvent extraction, steam extraction, supercritical fluids extraction, microwave-assisted extraction, subcritical water extraction or high hydrostatic pressure extraction (Starmans and Nijhuis, 1996; Stalikas, 2007; Zhang et al., 2011; Khoddami et al., 2013; Khoomrung et al., 2013). These methods often allow for the addition of internal standards (ISs) at the beginning of the extraction step to enable, e.g., accurate quantification of the metabolite of interest, and normalization against technical variability or other experimental variations. Furthermore, the addition of ISs is helpful for subsequently calculating the efficiency of extraction or purification of the clean-up methods. In addition to the extraction and quantification steps, the identification of metabolites is also a crucial step in metabolomics. This can be performed using two separate and complementary approaches: untargeted and targeted (Patti et al., 2012).

### Untargeted metabolomics

**Figure 1:**
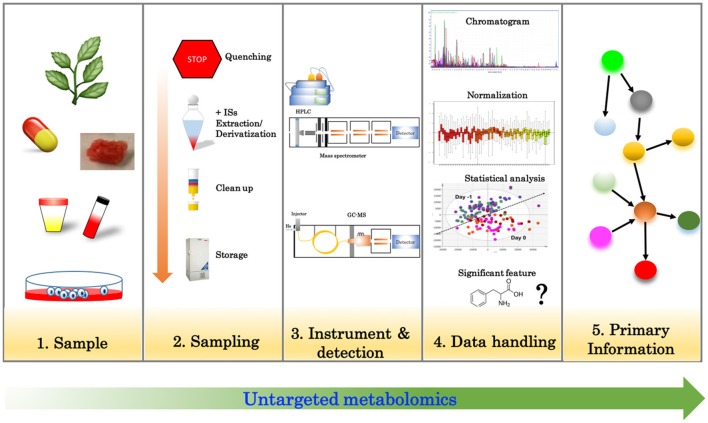
Overview of the steps involved in untargeted metabolomics.

Untargeted analysis (Figure 1) aims to simultaneously detect as many metabolites as possible in a given sample. Liquid or gas chromatography (LC/GC) coupled with mass spectrometry (MS) and nuclear magnetic resonance (NMR) are often employed for this purpose (Wishart, 2016). Untargeted metabolomics relies heavily on the technology for the measurement of numerous features (metabolites) as well as bioinformatics tools to handle the dataset. With the latest advancements in MS technology, it is now possible to routinely detect more than 2000 features in a single run; however, data processing, data analysis and metabolite identification remains a big challenge of this approach.

#### Analyzing untargeted MS-based metabolomics data

The ultimate goals of metabolomics data analysis are quantification and identification of compounds in the sample. The typical output from GC-MS and LC-MS are the chromatograms representing the amounts of detected compound(s), and mass spectra representing the fingerprint of the compound. Ideally, a chromatogram represents the amount of an individual compound. However, in many situations, co-elution of more than one compound is often encountered. Deconvolution (Colby, 1992) can be applied, which uses an algorithm to discriminate a desired signal from raw data. This is particularly useful when the desired signal has been contaminated by other interferences. This process can be performed using commercial software from different vendors such as, Agilent technologies, ThermoFisher, Waters Corporation, or from a research institution such as, the National Institute of Standards and Technology (NIST). Typically, the algorithm detects peaks by calculating signal to noise ratios, then performs calibrations using data from chromatograms and *m/z* aligment (Niu et al., 2014) to obtain confidence features and their intensity. Normalization and scaling are the next two steps to make data comparable across different samples.

Usually, a single IS or mix of ISs are used as a control to counter the unwanted variations which may occur during experimental measurements. It has been shown that statistical models utilizing multiple ISs for normalization often provide more robust results as compared to the use of a single IS (Redestig et al., 2009; Risso et al., 2014). Sysi-Aho et al. (2007) introduced a normalization method called normalization using optimal selection of multiple ISs (NOMIS) (Sysi-Aho et al., 2007). This method employs multiple ISs to estimate the optimal value for normalization of an individual feature in the chromatogram. Furthermore, both supervised and unsupervised approaches can be applied in the NOMIS algorithm. In 2009, Redestig and co-workers introduced a method called cross-contribution compensating multiple standard normalization (CCMN) (Redestig et al., 2009). This CCMN method was built on a supervised statistical model and aimed to capture the influence of all metabolites in the sample based on the signal of ISs. The method follows the concept of cross-contribution and uses the model to correct the unwanted variation. In De Livera et al. (2015) proposed a normalization method based on a linear mixed effects modeling approach. This algorithm performed well by being able to reduce a greater number of unwanted variations from datasets as compared to other methods such as, NOMIS or CCMN. A great attribute of all aforementioned algorithms here is that they have been implemented as packages in R suite software, which is freely available for researchers.

The next step in metabolomics data analysis is to find important features such as, significant metabolomic differences comparing between experimental conditions. A univariate analysis such as, the parametric *Student's t-test*, ANOVA or non-parametric Mann-Witney *U*-test is typically the first method of choices for the standard analysis. Multiple testing corrections such as, false discovery rate (FDR) can then be performed to balance the number of false positives and false negatives. When performing a univariate analysis, the most common output for results will be in the form of fold changes and *p-*values (adjusted-*p*-values). These results can be used to further evaluate the identities of significant features in the considered dataset. In untargeted metabolomics, it is common for more than two variables or features (e.g., metabolite identity, peak intensity, retention times or mass spectra) to be simultaneously measured in one sample. Therefore, multivariate analysis is used for capturing possible relationships between individual variables. Two well-known methods that have been used extensively are principal component analysis (PCA) and partial least square analysis (PLS). PCA (Wold et al., 1987) is an unsupervised method that is often used to evaluate intrinsic variability among the observations (samples). PCA is a dimensional reduction method that employs covariances of a dataset to transform the data to a new coordinate system, enabling one to distinguish which factors contribute most to the variability in the data. Mathematically, the number of principal components will be equal to the number of original samples. In PCA, the 1st component captures the largest possible amount of variance in the data, whereas the 2nd component describes the next-largest variation in a direction that is orthogonal to the 1st component. Unlike PCA, PLS is a supervised method that needs to have defined categorical variables. PLS applies multiple linear regression models by projecting measured variables to the categorical variables in a new space. The PLS model can further be used for discriminatory analysis referred to as PLS-DA (partial least square discriminant analysis). Typically, a PLS-DA model is formulated from the separation planes between classes (more than a 1-dimensional plane), leading to class-specific information being given by the PLS-DA model (Bylesjo et al., 2006). In Trygg and Wold (2002) proposed an extension of PLS-DA model by incorporating an orthogonal signal correction method which was previously proposed by Wold et al. (1998) called OPLS-DA (orthogonal partial least square discriminant analysis). Incorporation of orthogonal signal correction into PLS maximizes the explained covariance in the model, leading to improved discrimination among classes when compared with PLS-DA. Both PLS-DA and OPLS-DA can be performed in SIMCA (Soft Independent Modeling of Class Analogies) software (Bylesjo et al., 2006) or freeware under R suite environment (Thevenot et al., 2015). Multivariate analysis approaches are comprehensively reviewed by Worley et al. (Worley and Powers, 2013) and have been applied to various metabolomic studies, including the analysis of plant metabolomics (Madala et al., 2014; Hagel et al., 2015), plasma and serum metabolomics (Barri and Dragsted, 2013), and metabolomic data of lung cancer tissues (Wikoff et al., 2015).

### Targeted metabolomics

**Figure 2:**
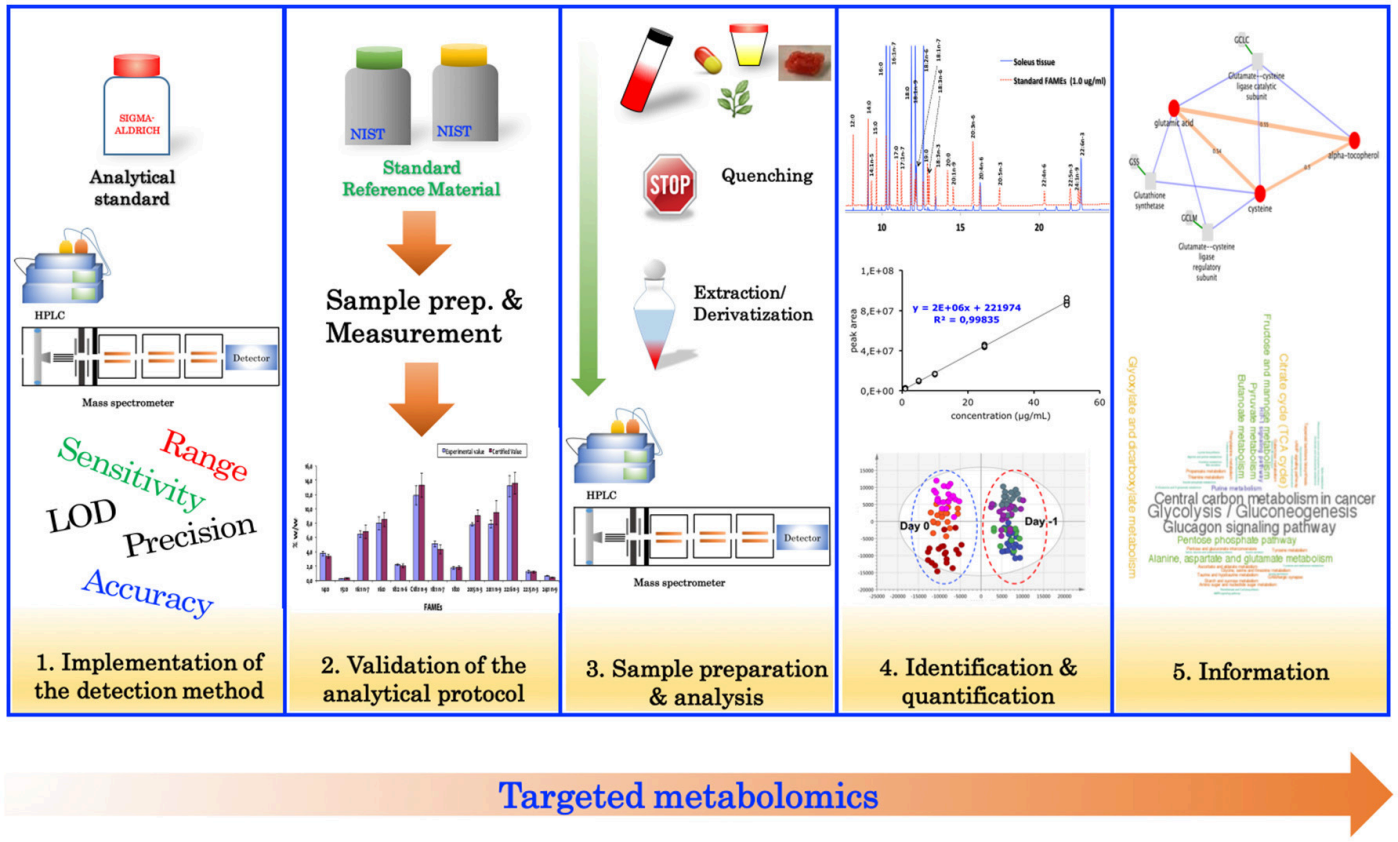
Overview of the steps involved in targeted metabolomics.

Targeted metabolomics relies heavily on analytical chemistry, and typically involves the measurement of specific metabolites of interest, or for confirmation of results from an untargeted analysis. Traditionally, the targeted approach (Figure 2) begins with the evaluation of the analytical performance of the detection method (instrument) such as, specificity, linearity, sensitivity, limit of detection, limit of quantification, accuracy, and precision. Typically, evaluating the performance of these parameters is performed using authentic standards. Once the detection method has been implemented, the analytical protocol (including sample preparation) should be validated. Validation of the analytical protocol is normally accomplished by using a standard reference material (Khoomrung et al., 2012, 2014; Phinney et al., 2013) (if available) or a spiking experiment to estimate the overall recovery of the protocol (Khoomrung et al., 2013). After validation of the analytical procedure, the method is then applied to quantify metabolites in the real samples of interest.

### Metabolome databases

Once the significant features have been identified from statistical methods, the next critical step is to determine their identity. This step is particularly challenging for untargeted metabolomics where metabolic identification is the largest bottleneck for acquiring new biological knowledge from the given study. Putative identification of the significant features obtained by MS are normally performed based on an accurate measurement of the mass-to-charge (*m/z)* ratio to define the molecular formula. Normally, mass spectra and/or retention time will be searched against MS libraries through databases; for example, metlin, lipidmaps, HMDB, or massbank (Tautenhahn et al., 2012), to identify possible compound name(s) for the queried MS peaks. The two problems most often encountered are either there are no hits, or multiple hits are found. The putatively identified metabolites are subsequently confirmed with an MS/MS experiment, by comparing their mass spectra and retention time with the authentic standards or an independent targeted experiment (Section Targeted Metabolomics). Even though metabolite identification remains a major challenge in untargeted metabolomics, there exists a wide variety of metabolome databases to assist with this process. The recent review by Fukushima and Kusano (2013) summarized a wide range of metabolome databases, which include mass spectrum-oriented databases for metabolite identification; compound-oriented databases for chemical information; metabolite-profile databases for compound information; and pathway-oriented databases for compound annotation in the context of metabolic pathways (Fukushima and Kusano, 2013). Further information on the strategies, approaches, and tools used for metabolite identification are beyond the scope of this review, and have been covered in greater detail by Dunn et al. (2013), Fukushima and Kusano (2013) and Vinaixa et al. (2016).

## Integrative omics data analysis for TTM

Single omics has until now been the predominant form of global data acquisition and analysis, with transcriptomics being one of the most mature and commonly studied omics (Ma et al., 2001; Wang et al., 2017; Yin et al., 2017). Transcriptomics provides a snapshot of mRNA profiles within a specific context, and is increasingly used in Chinese medicine research to characterize physiology, regulatory mechanisms and metabolisms of Chinese medicinal herbs, as reviewed in Lo et al. (2012). Another common omics method, proteomics, involves the quantification of proteins, protein post-translational modifications, and protein interactions. For instance, in the area of herbal medicine, proteomics has been used to investigate the mechanism of action of a Chinese herb, *Salvia miltiorrhiza* (Hung et al., 2010) and abundance of Ginseng peptides (Ye et al., 2016). Metabolomics data provide the comprehensive profiling of metabolites in a system under consideration. Typically metabolites, along with proteins, are the molecular components that ultimately carry out the cellular functions encoded by the genome. Hence metabolite levels can be considered as the most reflective of biological systems to any perturbations. There are a number of studies employing metabolite profiles to describe molecular mechanisms of medicinal plants (Fukuhara et al., 2011; Liu et al., 2013; Zhang et al., 2013; Le et al., 2016; Jiang et al., 2017). Additionally, metabolomics has been applied for drug discovery, pharmacokinetic analyses, pharmacodynamics investigations, evaluation of toxicity and toxic mechanisms of compounds in phamaceutical research (Zhang et al., 2014; Su et al., 2016; Cui et al., 2017; Kantae et al., 2017; Li et al., 2017). The emergence of metabolomics has enabled this type of data to be integrated with other omics types (genomics, transcriptomics, epigenomics, and proteomics) to gain a more comprehensive understanding of biological systems under study. However, translation of these omics data into biologically meaningful knowledge is still progressing and requires the development of more advanced computational and statistical methods. Currently, there exist several approaches for the integrative analysis of omic data which can potentially be applied to TTM. For example, in plant research, genome-scale metabolic models have been exploited in conjunction with metabolomics and transcriptomics to examine characteristics of metabolic networks, unravel metabolic phenotypes and further guide genetic engineering (Fukushima et al., 2014; Lakshmanan et al., 2015, 2016; de Oliveira Dal'Molin et al., 2016). Furthermore, a systems biology-based approach has been used to elucidate mechanisms of traditional Chinese medicinal formulas in specific diseases (Huang et al., 2015; Zhao et al., 2017).

Conceptually, methods which aim to integrate multi-omic data can be divided into two approaches: horizontal and vertical integration (Tseng et al., 2012). Horizontal data integration has been used extensively over recent years for microarray meta-analysis. This method combines a single level of omic data sets (e.g., transcriptomics) which are under similar conditions (Marot et al., 2009; Shen and Tseng, 2010; Tseng et al., 2012; Xia et al., 2013). Meanwhile, vertical data integration assimilates multi-level omic data sets (e.g., integration of transcriptomics and metabolomics) and is the predominant method used in systems biology to integrate data (Xia et al., 2013). The concept of vertical data integration is the main focus of this part of the review. A number of computational- and statistical-based approaches for vertical integrative analysis include empirical correlation-based analysis (CBA) (Wanichthanarak et al., 2015; Cavill et al., 2016), pathway- or ontology-based analysis (Wanichthanarak et al., 2015; Cavill et al., 2016), network-based analysis (Fukushima et al., 2014; Wanichthanarak et al., 2015) and machine learning approaches (Li and Ngom, 2015).

**Figure 3:**
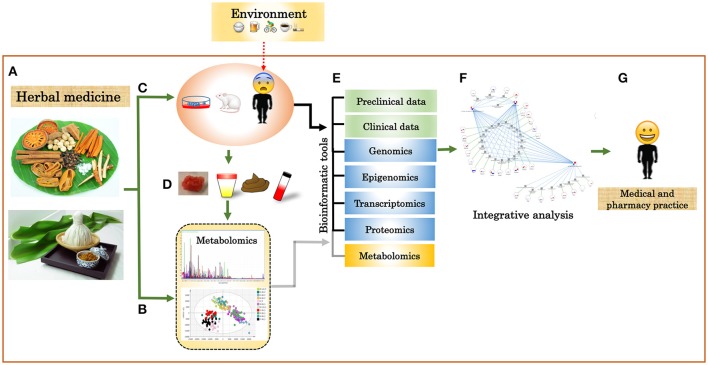
Integration of multi-omics and clinical data into TTM. **(A)** Herbal plants and herbal medicines. **(B)** Chemical characterization of herbal plants, herbal medicines, and biofluids using targeted and untargeted metabolomics. **(C)** Clinical trial of herbal plants and herbal medicines by cell lines, animal, or human models. **(D)** Biofluid for metabolome analysis. **(E)** Integrative analysis of different omics data and clinical data. **(F)** Systems biology network. **(G)** Individualized data.

For CBA, the primary aim is to find correlative links between data sets, such as, omic data and clinical data. In addition, CBA is useful for the analysis of unannotated metabolites from an untargeted analysis, since it has a limited number of biochemical domains or pathway information (Grapov et al., 2015). An effort to calculate a weighted correlation network of human blood metabolomics and transcriptomics has been demonstrated by Bartel et al. (2015). By creating this network, the authors were able to show that pairs of strongly correlated metabolites and transcripts biologically relate in terms of regulatory signaling and transport mechanisms. Pathway- or ontology-based analyses are one of the most commonly used methods for biological interpretations of omic data. It reduces data complexity by grouping related metabolites or genes based on pathways or biological functions before calculating enrichment statistics (Khatri et al., 2012). For example, a tool such as, IMPaLA (Integrated Molecular Pathway Level Analysis) improves identification of pathways through enrichment or overrepresentation analysis by integrating lists of metabolites and genes (Kamburov et al., 2011). The current generation of pathway analysis approaches includes additional information such as, pathway topology (Khatri et al., 2012; Li et al., 2013; Ihnatova and Budinska, 2015) and expression correlations (Feng et al., 2016) to enhance specificity, sensitivity and accuracy of pathway identification. From a network of molecular interactions, network-based analysis infers biological information from topological measures (such as, closeness centrality, degree distribution, degree centrality, betweenness centrality, and clustering coefficient (Winterbach et al., 2013). These biological networks can also serve as a scaffold for the integration of multiple omic datasets to identify active subgraphs, and support other visual analyses of omic data in the context of networks (Gerasch et al., 2014; Vehlow et al., 2015). A study by Fahrmann et al. uses a network derived from biochemical reactions and chemical structural similarity relationships to show metabolic alterations in type 1 diabetic and nondiabetic mice (Fahrmann et al., 2015). Machine learning approaches (such as, support vector machines, decision tree, Bayesian classifier, and neural networks) are capable of making predictive models and knowledge discovery by integrating data sets from several sources or multiple levels (Li and Ngom, 2015). Rajasundaram et al. (Rajasundaram and Selbig, 2016) summarized that integrative data analysis is often used for examining underlying relationships between omic datasets and for increasing predictive accuracy by using information from one or more datasets. One challenge of data integration is the heterogeneity of the different omics data. This can be considerably challenging for data-driven strategies, nonetheless applications of such methods are being increasingly reported in this big data era in various disciplines, such as, plant science (Bassel et al., 2011; Ma et al., 2014), chemoinformatics (Mitchell, 2014), drug discovery (Lavecchia, 2015), and medicine and health (Andreu-Perez et al., 2015; Kourou et al., 2015; Swan et al., 2015; Hao et al., 2016). It can therefore be seen that these methods can be successfully applied to study the properties and impacts of TTM. By intergating different omics as well as clinical data (both *in vitro* and *in vivo* experiments) for the study of TTM (Figure 3), it will therefore be possible to accelerate the development of TTM in several aspects such as, drug discovery and development, and medical treatment.

## Current metabolomics activities related to TTM

Metabolomics is a relatively new approach to the field of TTM, with only targeted approaches having been performed so far, particularly in the area of herbal plants. Most of the analyses have focused on searching for novel or known bioactive compounds from herbal plants. In many cases, the analysis of metabolites from the herbal plants was performed without a quenching step. The traditional liquid extraction remains the most popular method and is widely used in herbal plant research because of simplicity, easy to develop protocal, universal application, and relatively low cost. Sutthanut et al. (2007) employed ethanol (95%, v/v) to extract flavonoids from *Kaempferia parviflora* (Krachaidum). The rhizomes of Krachaidum have been widely used in TTM, especially for the treatment of health, promoting anti-inflammatory activity (Yenjai et al., 2004) or gastrointestinal disorders (Jaipetch et al., 1983). In 2014, Padumnonda and co-workers (Padumanonda et al., 2014) also reported the use of methanol to extract melatonin (N-acetyl-5-methoxytryptamine) from seven herbs. The seven samples used in this study were *Piper nigrum* L, *Sesbania glandiflora* (L.) Desv., *Sesbania sesban* (L.) Merr., *Senna tora* (L.) Roxb, *Moringa oleifera Lam., Momordica charantia* L. and *Baccaurea ramiflora* Lour. These herb samples are normally recommended for sleeping aids or treatment of insomnia.

In general, methanol and ethanol are the most widely used organic solvents for extraction of different metabolites from herbal plants, and the combination of methanol and water is also very attractive for many biological samples (Kanchanapoom et al., 2001; Villas-Bôas et al., 2006; Teo et al., 2011; Padumanonda et al., 2014). However, these extraction methods may not always provide relevant data for all cases in TTM, such as, in the case of water decoction (hot aqueous extract), whereby a liquid medicine is made from the extraction of multiple herbs in boiling water. After the medical preparation, only the liquid fraction is used as the medicine and administered to patients. The goal of metabolome analysis in this scenario is to chemically characterize metabolites present in the extract taken by the patients, rather than the entire set of metabolites contained within the herbs. In this case, water would be a more appropriate solvent for extraction as compared to methanol or ethanol. Furthermore, the downstream protocol used for the chemical analysis should be prepared as similarly as possible to the preparation process of the decoction medicine (glassware, time, and temperature). This will improve the accuracy of the metabolome information, and will greatly affect the overall outcome of the study. On the other hand, analysis of herbal medicines or herbal products can be even more complicated (Charoonratana et al., 2014). For example, many Thai medicines result from the mixture of different herbal material in various quantities to one another. These mixtures are ultimately manufactured in different forms; for example, in pill, capsule, or bolus form, and are consumed directly by the patient, without using an extraction step. However, the chemical compositions of these drugs are largely unknown, and a chemical analysis using a traditional extraction such as, a single solvent or two-phase solvent systems (e.g., methanol-water, methanol-chloroform) will yield an incomplete picture of the drug composition, especially if only one fraction is analyzed. This is therefore unlikely to provide sufficient information about all the possible metabolites that have been consumed by the patients, as there is no universal extraction method that can cover the entire spectrum of metabolites in a single extraction. In such cases, a good extraction protocol for chemical analysis of herbal medicines may include using more than just one solvent, or the extraction protocol can be performed more than once with different solvents (Yang et al., 2016). Increasing proportion of the organic solvents in the extraction process can lead to an increase in the number of extracted metabolites; however, this may require many additional sample clean-up steps prior to the measurements. In a previous study by the Nielsen group (Khoomrung et al., 2015), it was shown that the coverage of metabolites in a yeast sample can be increased (by 16%) when the analysis is performed on both polar and non-polar fractions as compared to the results from the polar-fraction alone. This approach could potentially be adopted to analyze the metabolome following treatment with herbal medicines. A study by Teo et al. (2011) demonstrated the use of multiple approaches for global metabolome analysis in *Stevia rebaudiana* and *Coptidis rhizoma*. The authors used methanol-water extraction and green-solvent microwave-assisted extraction (MAE) for the extraction of primary and secondary metabolites, and the subsequent isolated-extracted metabolites were monitored with GC-MS, ^1^H NMR and HPLC-UV techniques. Analysis of primary metabolites from the methanol fraction by GC-MS and ^1^H NMR in both samples showed the presence of the common polar and slightly non-polar metabolites such as, amino acids, sugars, organic acids, carbohydrates, and lipids. Analysis of the secondary metabolites by HPLC-UV after MAE extraction showed fewer metabolites e.g., stevioside, rebaudioside and berberine. The profiling of primary metabolites enabled a clear differentiation of sample origins, whereas metabolome data from secondary metabolites could not be used to distinguish samples from different sources.

In contrast to the targeted approach, the development and application of untargeted metabolomics in TTM is relatively unexplored. This is in part due to the relatively recent emergence of metabolomics, as well as the ongoing advancement of technologies such as, GC-MS, LC-MS, NMR, and the pace of advancement in bioinformatics for processing big data in the area of TTM. Nonetheless, due to the rapid evolution of these technologies and approaches, it is expected for progress to advance quickly in the early stages of development.

## Potential applications of metabolomics to TTM

In the early stage of development, an application of metabolomics to TTM could potentially be seen in the area of medical and pharmacy practice. It is now widely accepted that metabolite levels are highly sensitive to environmental or physiological changes; thus, metabolomics could serve as an excellent tool to characterize metabolic profiles to help diagnose and treat patients. This could easily be accomplished through a minimally invasive, untargeted approach, e.g., analyzing body fluids such as, serum, plasma, urine, and saliva from subjects with different illnesses and comparing the results with healthy subjects. For example, the broad spectrum of detected features (metabolites) could aid the discovery of significant molecules contributing to particular diseases. Subsequently, such compounds can be validated using the targeted approach, which is more sensitive and accurate in determining both identity and quantity of a given metabolite. These strategies have been successful in the diagnosis of several diseases in modern medicine (Armitage and Barbas, 2014). Another promising application of metabolomics would be the use of targeted metabolomics to characterize chemical structures and components of bioactive compounds in herbal medicines, since one of the bottlenecks in herbal medicines is that most of the active components of the drugs are poorly defined. Furthermore, the constituents of these compounds can vary greatly from batch to batch, depending on several factors; for example, age of the plant, cultivation conditions (weather and season) or production process. In this context, the targeted metabolomics approach is particularly useful, by characterizing and defining the exact identities and concentrations of bioactive compounds contained within herbal material for each given product. This information would assist greatly in controlling the consistency of herbal medicine produced from different batches. Furthermore, understanding the chemical composition of herbal medicines provides an opportunity to elucidate their mechanism of action after taken by the patients. Untargeted metabolomics could be performed to profile metabolites from biofluids after the patients have taken the drugs, whereas targeted analysis will assist in the elucidation of metabolic processing of an individual drug. This information is particularly useful to aid the design and development of herbal medicine as well as to improve treatment and therapy in TTM.

## The establishment of a national infrastructure to improve TTM

At the Faculty of Medicine Siriraj Hospital, Mahidol University, we have been conducting research in several areas of systems biology and systems medicine such as, genomics, proteomics, metabolomics, and clinical research. To further increase research capacity, we have recently established the university metabolomics and phenomics platform, namely Siriraj Metabolomics and Phenomics Center (SiMPC) at Mahidol University. The aim of the SiMPC is to develop a top-level university platform for metabolomics and phenomics research. The center will conduct both research and service in the fields of metabolomics and phenomics as well as providing education and training to the students and researchers in Thailand. The scope of diseases and research topics will focus initially on diabetes mellitus, cancer, cardiovascular disease, dengue hemorrhagic fever, transplantation, and TTM. Using an integrated big data network at Siriraj Hospital, this will lead to the development of precision medicine and facilitate the translation of scientific knowledge into clinical practice for high impact diseases.

## Conclusion and perspectives

TTM is a holistic medicine with a long history of serving Thai society for many generations. Furthermore, TTM has remained a highly popular medical resource in Thailand for the Thai community; however, a major drawback of their use, until now, has been a lack of clinical evidence for what bioactive compounds they contain that mediate their therapeutic effects, and what their mechanism of action is within the patient. To promote the use of TTM in society, metabolomics can be utilized to identify chemical compositions, to screen for potentially active compounds in herbal plants or herbal medicines, as well as to evaluate the therapeutic effect of TTM during pre-clinical and clinical studies. Furthermore, integration of metabolomics and other omics will help to improve the overall understanding of the phenotypic characteristics of an individual subject, and will increase the rate of TTM development. Since metabolomics is relatively new to TTM, perhaps the greatest challenge in the early stages of development would be in implementing a measurement technology, such as, routine protocols for untargeted and targeted analysis, alongside the bioinformatics platform to handle the large dataset subsequently produced. Infrastructures, such as, SiMPC, will thus provide a promising contribution toward efforts to accelerate the development of TTM in the future.

## Ethics statement

This article does not contain any studies with human participants or animals performed by any of the authors.

## Author contributions

SK, IN, and KW designed and drafted the manuscript. OT, PS, TL, and PA helped to draft the manuscript. SK, IN, KW, and PA made critical revisions to the final version. All authors reviewed and approved the final manuscript.

### Conflict of interest statement

The authors declare that the research was conducted in the absence of any commercial or financial relationships that could be construed as a potential conflict of interest.
